# Effects of tamoxifen on vaginal blood flow and epithelial morphology in the rat

**DOI:** 10.1186/1472-6874-6-14

**Published:** 2006-09-13

**Authors:** Noel N Kim, Miljan Stankovic, Abdullah Armagan, Tulay T Cushman, Irwin Goldstein, Abdulmaged M Traish

**Affiliations:** 1 Institute for Sexual Medicine, Department of Urology, Boston University School of Medicine, Boston, Massachusetts, USA; 2 Department of Urology, Suleyman Demirel University Faculty of Medicine, Isparta, Turkey; 3 Department of Anatomy and Neurobiology, Boston University School of Medicine, Boston, Massachusetts, USA; 4 Department of Biochemistry, Boston University School of Medicine, Boston, Massachusetts, USA

## Abstract

**Background:**

Tamoxifen, a selective estrogen receptor modulator with both estrogenic and anti-estrogenic activity, is widely used as adjuvant therapy in breast cancer patients. Treatment with tamoxifen is associated with sexual side effects, such as increased vaginal dryness and pain/discomfort during sexual activity. There have been limited investigations of the effect of tamoxifen on estrogen-dependent peripheral genital arousal responses. The objective of this study was to investigate the effects of tamoxifen on vaginal physiology in the rat.

**Methods:**

Female Sprague-Dawley rats were subjected to sham surgery or bilateral ovariectomy. After 2 weeks, sham-operated rats were implanted with subcutaneous osmotic infusion pumps containing vehicle (control) or tamoxifen (150 μg/day). Ovariectomized rats were similarly infused with vehicle. After an additional 2 weeks, vaginal blood flow responses to pelvic nerve stimulation were measured by laser Doppler flowmetry and vaginal tissue was collected for histological and biochemical assay.

**Results:**

Tamoxifen treatment did not change plasma estradiol concentrations relative to control animals, while ovariectomized rats exhibited a 60% decrease in plasma estradiol. Tamoxifen treatment caused a significant decrease in mean uterine weight, but did not alter mean vaginal weight. Vaginal blood flow was significantly decreased in tamoxifen-infused rats compared to controls. Similar to ovariectomized animals, estrogen receptor binding was increased and arginase enzyme activity was decreased in tamoxifen-infused rats. However, different from control and ovariectomized animals, the vaginal epithelium in tamoxifen-infused rats appeared highly mucified. Periodic acid-Schiff staining confirmed a greater production of carbohydrate-rich compounds (*e.g. *mucin, glycogen) by the vaginal epithelium of tamoxifen-infused rats.

**Conclusion:**

The observations suggest that tamoxifen exerts both anti-estrogenic and pro-estrogenic effects in the vagina. These physiological alterations may eventually lead to vaginal atrophy and compromise sexual function.

## Background

Tamoxifen is a non-steroidal, triphenylethylene derivative that is widely used and highly effective as adjuvant therapy for the management of breast cancer patients with estrogen receptor (ER) positive tumors. Originally developed in the 1960s as a contraceptive agent, tamoxifen was later found to stimulate ovulation, as well as inhibit the formation of mammary tumors in rats exposed to carcinogens [1]. While tamoxifen itself is a poor ER ligand, its metabolite 4-hydroxytamoxifen is a potent antagonist with high affinity for the ER [2,3]. In addition, the effects of tamoxifen can be stereo-selective. Trans-tamoxifen is generally considered an anti-estrogen, whereas cis-tamoxifen is considered to be a weak estrogen. Interconversion between these isomers has been shown to occur in cultured cells [4], but this process remains poorly characterized *in vivo*.

Acknowledged as a first generation selective ER modulator (SERM), tamoxifen and/or its metabolites exert anti-estrogenic effects in breast tissue, estrogenic effects on bone (increased density) and plasma cholesterol (decreased levels), and mixed effects in the uterus [1,5,6]. In addition to the beneficial effects of tamoxifen, its complex, tissue-specific action can also result in adverse effects in women. Peri-menopausal symptoms of hot flushes and night sweats are frequently reported side effects in both pre- and post-menopausal women on tamoxifen therapy [5-8]. In pre-menopausal women, tamoxifen has also been reported to cause oligomenorrhea or amenorrhea with a return to normal menses after discontinuing tamoxifen [9]. Yet, the greatest concern has been focused on the effects of tamoxifen in the uterus. Tamoxifen treatment increases the relative risk for endometrial cancer [10-12] and may increase the incidence of endometriosis and uterine mesenchymal (*i.e. *myometrial) tumors [6].

While the psychological impact of breast cancer diagnosis and subsequent treatment can cause profound changes in sexual function, tamoxifen itself may increase the incidence of sexual dysfunctions. Limited studies indicate that tamoxifen treatment is associated with decreased sexual interest/desire and increased vaginal dryness and tightness [6,8,13,14]. In a study designed to specifically assess the effects of tamoxifen on sexual function in women, 22 of 41 sexually active subjects (54%) experienced pain/discomfort during intercourse that was unrelated to previous chemotherapy [7]. The duration of tamoxifen treatment ranged from 2 to 24 months with a median duration of 12 months.

Thus, breast cancer patients treated with tamoxifen as adjuvant therapy may exhibit symptoms of estrogen deprivation, including sexual dysfunction related to alterations in vaginal tissue structure and function. Sexual health remains an important contributor to quality of life in breast cancer survivors [15]. The use of tamoxifen as a prophylactic agent in younger, pre-menopausal women at risk for breast cancer is expected to increase. Since younger women may be exposed to tamoxifen for longer periods of time, determination and consideration of adverse side effects will be important. In laboratory animal studies that simulate sexual arousal through activation of pelvic parasympathetic nerves, we and others have previously demonstrated that the vaginal responses of increased blood flow and fluid transudate production are estrogen-dependent [16-19]. Given the well-acknowledged estrogen dependency of genital tissues, the preliminary clinical association of tamoxifen treatment with sexual dysfunction in women, and the critical role of estradiol in maintaining the vaginal response to sexual arousal, we investigated the effects of tamoxifen on vaginal blood flow and tissue morphology in the rat. We further assessed changes in ER expression and arginase activity as estrogen-dependent markers in the vagina.

## Methods

### Animals

Mature female Sprague-Dawley rats (strain Crl:CD; 8–10 weeks old; 200–225 g) were purchased from Charles River Laboratories (Wilmington, MA) and used for investigation. These studies were approved by the Institutional Animal Care and Use Committee at the Boston University School of Medicine. Animals were subjected to sham surgery or bilaterally ovariectomized. After 2 weeks, sham-operated rats were implanted with subcutaneous osmotic infusion pumps (model 2002; Alzet, Palo Alto, CA) containing polyethylene glycol 300 (vehicle-infused control group; CONT, n = 11) or tamoxifen released at a rate of 150 μg/day (TMX, n = 11). Ovariectomized rats (OVX, n = 8) were similarly infused with vehicle. Two weeks after pump implantation, *in vivo *studies were carried out to assess vaginal blood flow, as described below.

### Assessment of estrus cycle phase

For all animals, smears of vaginal cells were prepared before vaginal blood flow measurements. A cotton swab moistened in 0.9% saline was inserted into the vagina, manipulated in a circular fashion and then smeared onto a glass slide. Samples were fixed with 70% ethanol and stained with hematoxylin and CytoQuik stain (Fisher Scientific). The phase of the estrous cycle was determined from the morphological characteristics of the vaginal cells. All samples were examined by a licensed cytologist.

### In vivo assessment of vaginal blood flow

Changes in vaginal blood flow in response to pelvic nerve stimulation were determined by laser Doppler flowmetry, as previously described [20]. Briefly, rats were anesthetized with intramuscular injections of ketamine (40 mg/kg) and xylazine (4 mg/kg). To continuously monitor systemic blood pressure, a 24-gauge angiocatheter was introduced into the carotid artery and connected to a pressure channel of the laser Doppler flowmeter (Model BLF21D, Transonic Systems, Inc., Ithaca, NY). A surface probe (Type I; Transonic Systems, Inc.) was placed in the vaginal canal, facing the anterolateral vaginal wall and approximately halfway between the introitus and the cervix. Unilateral pelvic nerve stimulation (PNS) was accomplished with a Grass S9 stimulator set at normal polarity and repeat mode to generate a 30 second train of square waves with 6 Volt pulse amplitude and 0.8 millisecond pulse duration at various frequencies (5, 10, 20 Hz). Blood flow data were recorded as tissue perfusion units (TPU). Hemodynamic parameters (vaginal blood flow and systemic blood pressure) were continuously recorded (collection rate = 100 Hz) throughout the experiment using WinDaq software (version 2.15; Dataq Instruments, Akron, OH). Since blood flow through any given tissue is dependent upon the systemic blood pressure, we also calculated the instantaneous vascular resistance (systemic blood pressure/blood flow) at each time point. Using the vascular resistance plots, the area under the curve (AUC) for each response was determined.

### Determination of plasma estradiol concentration

Blood was drawn at the end of the vaginal blood flow studies. Plasma samples were sent to the Endocrinology Laboratory, Animal Health Diagnostic Center at the Cornell University College of Veterinary Medicine (Ithaca, NY) for determination of estradiol concentration. A solid-phase radioimmunoassay (RIA) with Coat-A-Count reagents (Diagnostic Products Corp., Los Angeles, CA) was used. The lower detectable limit of this assay was 8 pg/ml. Across a wide range of concentrations in the linear range, the inter-assay variation was 4–8% and the intra-assay variation of replicate samples was 4–7%. The most significant potential cross-reactants were estrone (10%), estrone-β-D-glucuronide (1.8%), estrone-3-sulfate (0.58%), estriol (0.32%), and estriol-3-glucuronide (0.012%). All other steroidal compounds, including tamoxifen, had cross-reactivity that was not detectable or extremely low (≤0.006%).

### Tissue procurement

After the completion of the *in vivo *studies, animals were exsanguinated and vaginal and uterine tissues were removed in their entirety, weighed and placed in chilled physiological salt solution. For histological analyses, vaginas from each treatment group (n = 5 per group) were opened with a longitudinal incision and a small notch was cut at the distal end for proper orientation. Tissues were then immersed in 10% neutral buffered formalin and stored at 4°C for 24–72 hours. For biochemical studies, vaginal tissues from 6 animals per treatment group (CONT and TMX) were frozen in liquid nitrogen and stored at -80°C until assayed. Due to the lower number of ovariectomized animals and the smaller amounts of tissue obtained from this group, a second set of intact controls and ovariectomized rats (n = 6 per group) were generated specifically for the biochemical assays.

### Preparation of cytosolic and nuclear extracts from vaginal tissue

Frozen vaginal tissue was pulverized with a Bessman tissue pulverizer (Spectrum Laboratories, Rancho Dominguez, CA) cooled on dry ice. Tissue powder from each treatment group was pooled and combined (1 g/4 ml) with ice cold buffer (20 mM HEPES, pH 7.4, 1 mM EDTA, 0.25 M sucrose). After the addition of phenylmethylsulfonylfluoride (PMSF; 0.5 mM) and mammalian protease inhibitor cocktail (1 mM AEBSF, 0.08 μM aprotinin, 20 μM leupeptin, 40 μM bestatin, 15 μM pepstatin A and 14 μM E-64; Sigma Chemical Co., St. Louis, MO), tissue powder was homogenized on ice using a Brinkmann PT3000 polytron with 10 second bursts and 30 second cooling intervals. The homogenate was centrifuged at 3000 × g for 20 min at 4°C and the supernatant (cytosol) was transferred to a fresh tube. An aliquot was used to determine soluble protein concentration and the rest of the cytosolic extract was frozen at -20°C until further assay. The pellet containing the nuclei was resuspended in ice cold buffer (50 mM Tris-HCl, pH 7.4 at 22°C, 1.5 mM sodium EDTA, 10% glycerol, 10 mM sodium molybdate and 10 mM monothioglycerol) containing 0.4 M KCl and homogenized on ice with a Dual glass-glass homogenizer using a motor driven pestle. The nuclear homogenate was incubated on ice for 1 hour and centrifuged at 100,000 × g for 30 minutes. The resulting supernatant (nuclear extract) was transferred to a new tube and frozen at -20°C.

### Receptor binding assay

Prior to performing the binding assay, free steroid hormone was removed from vaginal tissue extracts by incubating samples with dextran coated charcoal (DCC). An aliquot of DCC suspension (5 g acid washed charcoal and 0.5 g dextran in 1 liter of 10 mM Tris, pH 7.4 at 2°C, 1.5 mM EDTA, 0.02% NaN_3_) was placed in a tube and centrifuged for 10 min at 1000 × g. The supernatant was aspirated and an equal volume of cytosol/nuclear extract mixture was added to the DCC pellet. Samples were incubated on ice for 20 min with intermittent mixing and DCC was removed by centrifugation. Binding studies were carried out as described previously by Traish *et al*. [21]. Briefly, aliquots of the DCC-treated vaginal tissue extracts (100–200 μl) were mixed with an equal volume of buffer containing [2,4,6,7,16,17-^3^H(N)]estradiol (specific activity = 110 Ci/mmol; Perkin-Elmer Life and Analytical Sciences, Boston, MA) without (total binding) or with a 200-fold molar excess of unlabeled diethylstilbestrol (non-specific binding). Samples were maintained for 24 h at 2°C, followed by incubation at 25°C for 2 h and then re-incubated at 2°C for 22 h to measure occupied and unoccupied binding sites (exchange assay) [22]. The protein-bound radioactivity was separated from free radioactivity by hydroxylapatite assay [21].

### Arginase assay

Cytosolic vaginal tissue extract was used to determine arginase activity, as previously described [23]. Briefly, 10 μl of tissue extract (triplicate aliquots) were incubated in buffer (75 mM glycine, pH 9.0, 0.25 mM MnCl_2_) containing 300,000 dpm of [^14^C-guanidino]-L-arginine (51.5 mCi/mmol; NEN Life Science Products, Boston, MA), 4 mM of unlabelled L-arginine in a final volume of 100 μl. Samples were incubated for 60 min at 37°C and reactions were terminated by the addition of 400 μl of 0.25 M acetic acid (pH 4.5), 7 M urea, 10 mM L-arginine. After the addition of 500 μl of water, samples were passed through a 0.5 ml column of Dowex 50W-X8 resin (Bio-Rad Laboratories, Hercules, CA). Tubes were rinsed twice with 500 μl of water and both rinses were poured onto the columns. Columns were washed with an additional 1 ml of water and all effluent was collected in 20-ml vials. After the addition of 16 ml of Liquiscint (National Diagnostics, Atlanta, GA), radioactivity was quantified by liquid scintillation spectroscopy. Urea production was normalized to total soluble protein in the tissue.

### Histology

After fixation, the mid-section of each vagina was embedded in paraffin and subsequently sectioned at 5 microns using a rotary microtome. Tissue sections were deparaffinized with CitriSolv (Fisher Scientific, Pittsburgh, PA), rehydrated in graded ethanol solutions (100–70%), and subjected to a modified Masson's trichrome and periodic acid-Schiff (PAS) staining procedure, as previously described [24].

### Data analysis

The results were expressed as mean ± S.E.M. Comparisons between treatment groups were analyzed by unpaired t-tests or analysis of variance (ANOVA) followed by post-hoc comparisons to control (Dunnett's test), as appropriate. Means were considered significantly different if the p-value was less than 0.05.

## Results

**Figure 1:**
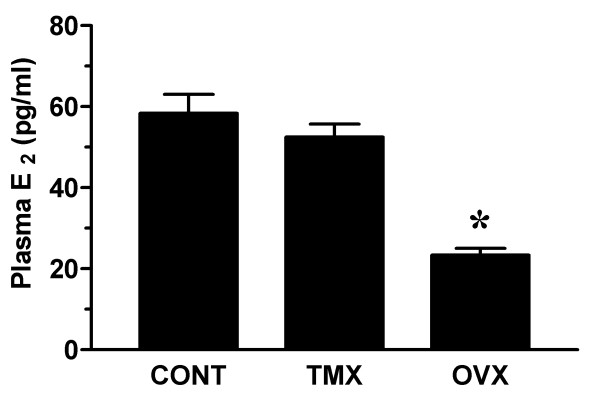
Plasma estradiol measurements. Female Sprague-Dawley rats were subjected to sham surgery or bilateral ovariectomy. After 2 weeks, sham-operated rats were implanted with subcutaneous osmotic infusion pumps containing vehicle (Control; CONT) or tamoxifen (TMX; 150 μg/day). Ovariectomized rats (OVX) were similarly infused with vehicle. After an additional 2 weeks, vaginal blood flow studies were performed and plasma samples from control (n = 11), tamoxifen-infused (n = 11) and ovariectomized (n = 8) rats were assayed for estradiol (E_2_) levels by conventional radioimmunoassay. Data are the mean ± SEM and were analyzed by one-way analysis of variance (ANOVA). *p < 0.05 versus control group.

**Figure 2:**
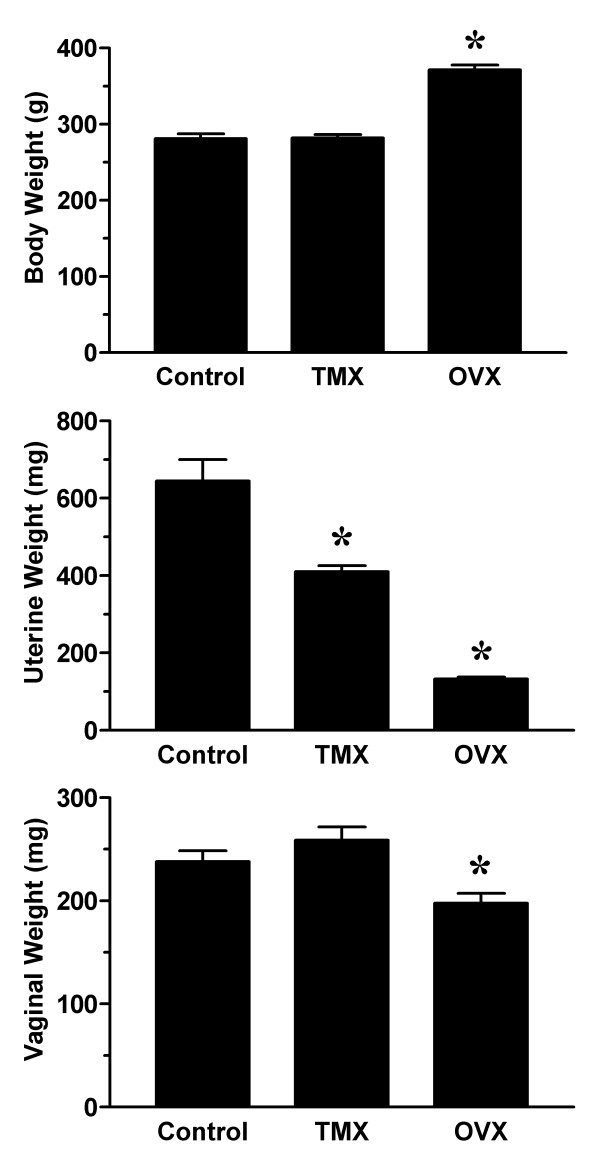
Effect of tamoxifen on body and tissue weights. Body weights were determined on the same day as vaginal blood flow studies in control (CONT), tamoxifen-infused (TMX) and ovariectomized (OVX) female rats (see Figure 1). Uterine and vaginal tissue wet weights were determined immediately following the blood flow studies. Data are the mean ± SEM and were analyzed by one-way ANOVA. *p < 0.05 versus control group.

Mean plasma estradiol concentration remained unaltered in tamoxifen treated rats, whereas a significant reduction in plasma estradiol was detected in ovariectomized rats (figure 1). Examination of vaginal smears indicated that all tamoxifen-infused and ovariectomized rats were in diestrus. In contrast, roughly half (5/11) of the rats in the control group were in diestrus with 2 each in proestrus, estrus and metestrus. Minimal changes in body weight were observed in rats treated with tamoxifen for two weeks (289 ± 4 g) when compared to age-matched controls infused with vehicle (281 ± 6 g) (figure 2). In contrast, ovariectomized rats were significantly heavier (371 ± 6 g). To assess the extent of anti-estrogenic effects of tamoxifen, the wet weights of the uterus and vagina were determined for each animal. As shown in figure 2, mean uterine wet weight was significantly lower in the tamoxifen treatment group (410 ± 15 mg) than the control group (644 ± 55 mg), but higher than that of ovariectomized animals (133 ± 4 mg). Interestingly, mean vaginal wet weight was similar in control and tamoxifen-infused rats, while a small but significant decrease was observed in ovariectomized rats.

**Figure 3:**
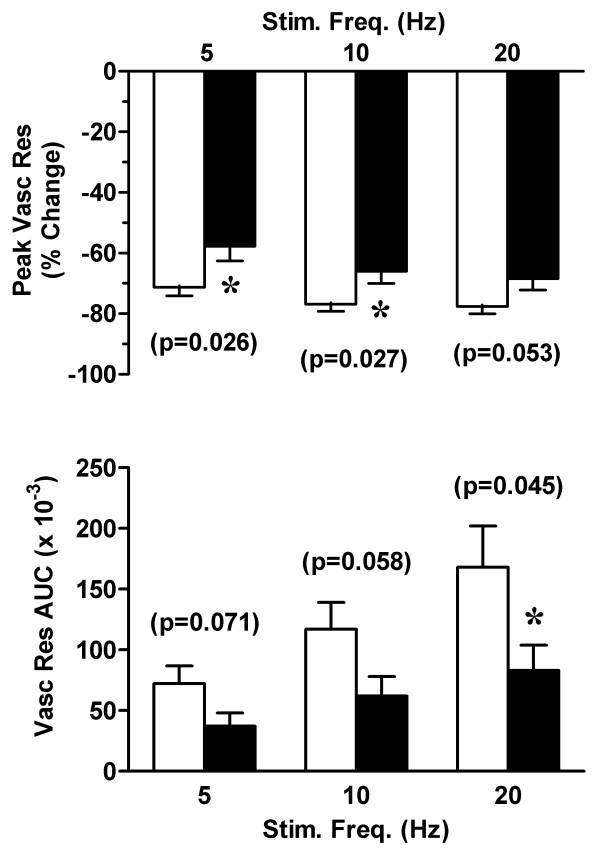
Effect of tamoxifen on vaginal blood flow. In control (empty bars) and tamoxifen-infused (solid bars) rats (see Figure 1), changes in vaginal blood flow were measured by laser Doppler flowmetry. Responses to pelvic nerve stimulation at varying frequencies (5–20 Hz) are expressed as the percent change in peak vascular resistance (relative to baseline values) and the area-under-the-curve (AUC). Data are the mean ± SEM and were analyzed by two-way ANOVA. *p < 0.05 versus control group.

*In vivo *studies in anesthetized rats demonstrated frequency-dependent increases in vaginal blood flow in response to pelvic nerve stimulation. While the percent reduction in peak vascular resistance was similar at all frequencies of stimulation, the change in total vascular resistance (AUC) was proportional to the stimulation frequency (figure 3). Consistent reductions in both the percent change in vascular resistance and the area under the vascular resistance curve indicated that vaginal blood flow was significantly decreased in tamoxifen-infused rats (figure 3).

**Figure 4:**
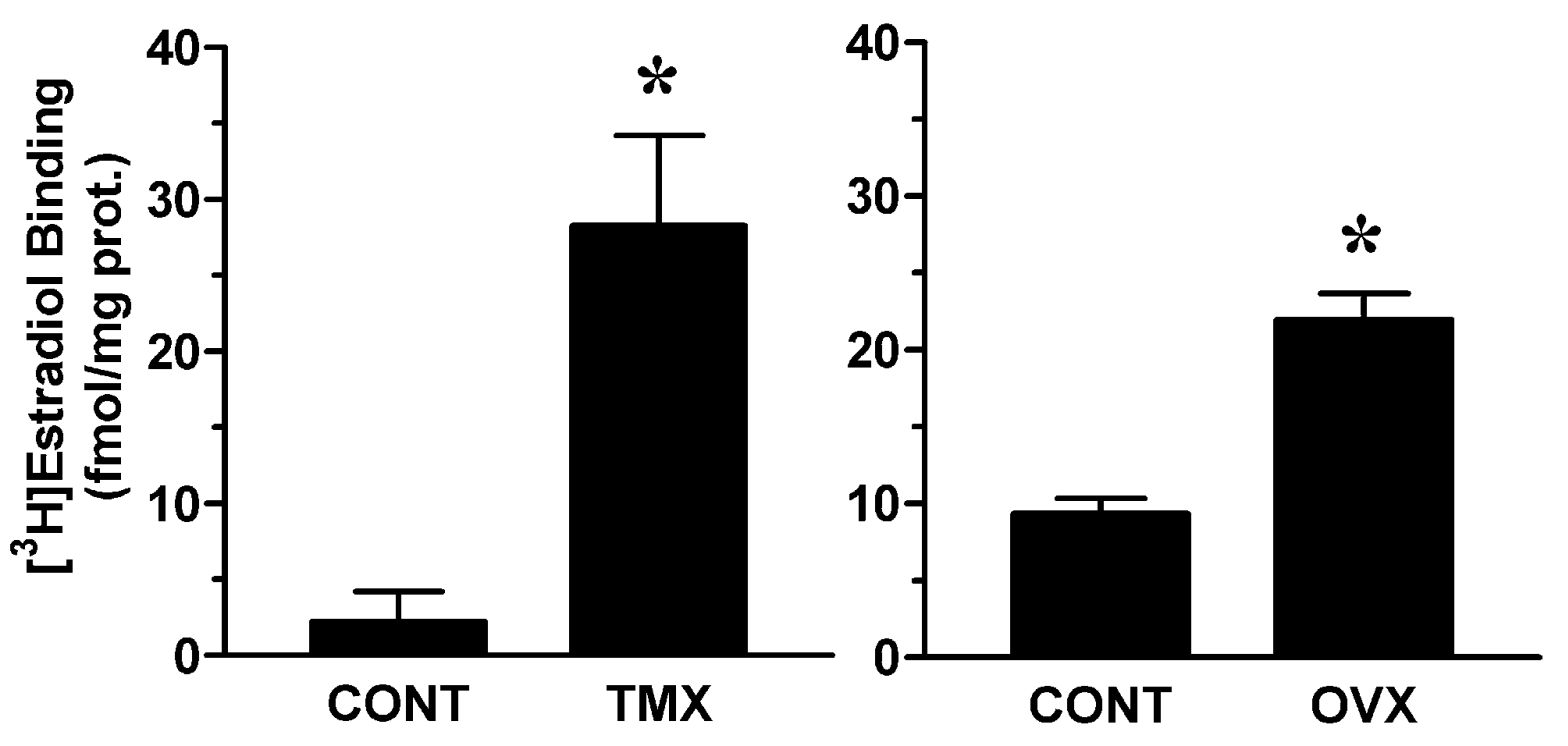
Effect of tamoxifen on vaginal estradiol receptor concentration. Total estradiol receptor concentration was determined by radioligand binding assay in vaginal tissue extracts from control (CONT) and tamoxifen-infused (TMX) rats (see Figure 1). For relative comparison, estradiol binding in vaginal tissue extracts from control and ovariectomized (OVX) rats was determined in separate assays. Data are the mean ± SEM from 3 independent assays. *p < 0.05 versus control group.

**Figure 5:**
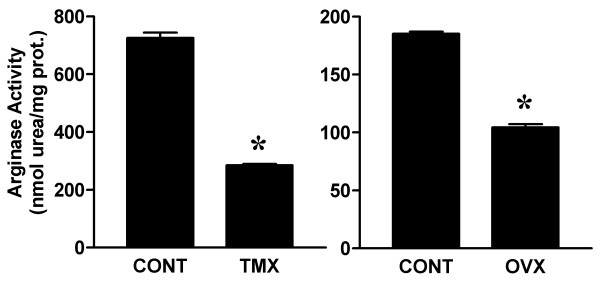
Effect of tamoxifen on vaginal arginase activity. Total arginase enzyme activity was determined in vaginal tissue extracts from control (CONT) and tamoxifen-infused (TMX) rats (see Figure 1). For relative comparison, estradiol binding in vaginal tissue extracts from control and ovariectomized (OVX) rats was determined in separate assays. Data are the mean ± SEM from 3 independent assays. *p < 0.05 versus control group.

To further characterize the effects of tamoxifen in the vagina, we determined ER concentration and arginase enzyme activity in vaginal tissue extracts. Tamoxifen treatment increased ER levels in the vagina and decreased arginase activity (figures 4 &5). Similar changes in ER binding and arginase activity were observed in vaginal tissue extracts of ovariectomized rats.

**Figure 6:**
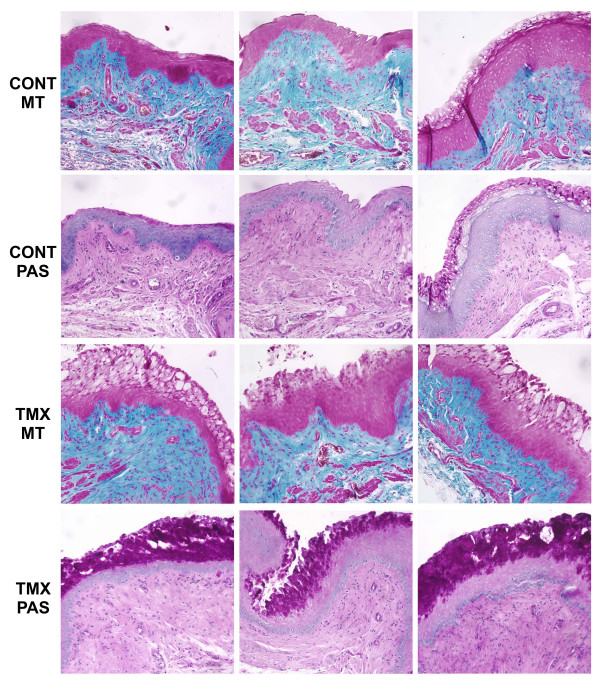
Effect of tamoxifen on vaginal epithelium. Vaginal tissue from control (CONT) and tamoxifen-infused (TMX) rats (see Figure 1) were fixed in 10% neutral buffered formalin and embedded in paraffin. Deparaffinized tissue sections (5 μm) were subjected to Masson's trichrome (MT) and periodic acid-Schiff (PAS) staining procedures. For each row, representative tissue sections from different animals within each treatment group are shown.

Examination of fixed vaginal tissue sections revealed normal variations in epithelial morphology in intact, cycling rats. These variations were most apparent at the apical surface of the epithelium and consisted of slightly keratinized, non-keratinized or mucified cells. Mucified epithelium was characterized by the presence of secretory vesicles and positive PAS staining (figure 6). A dramatic increase in the appearance and extent of mucified epithelium was observed in vaginal tissue from tamoxifen-treated animals. In addition, this mucified epithelial morphology was observed more frequently in tamoxifen-infused rats compared to vehicle-infused controls. No overt differences were noted in the lamina propria and muscularis layers when comparing vaginal tissue from control and tamoxifen-treated animals.

## Discussion

Sex steroid hormones play a critical role in the development and maintenance of female genital tissues. Despite this widely accepted perspective, characterization of vaginal responsiveness to steroid hormones in the adult has been limited mainly to the epithelial layer. However, several studies in animal models have demonstrated that fluctuations in ovarian hormones cause measurable changes in overall vaginal wall thickness, nerve density and structural organization of the muscularis layer [24-30]. Biochemical and functional *in vivo *studies suggest that estrogens are critical in maintaining vaginal blood flow, and fluid transudate production [16,17,19,31-33].

In the present study, the tamoxifen-induced changes in vaginal blood flow, ER expression and arginase activity were similar to those in ovariectomized animals and markedly differed from the control group. The trophic response of the uterus is a sensitive parameter to assess estrogenic effects. The significant decrease in uterine wet weight of tamoxifen-infused rats suggests that the dose of tamoxifen used (150 μg/day) was physiologically relevant and effective as an anti-estrogen to inhibit uterine growth. Tamoxifen treatment significantly reduced vaginal blood flow responses. This is consistent with our previous studies in which ovariectomized rats also exhibited a decrease in vaginal blood flow responses [19]. Thus, we attribute the decreased vaginal blood flow in tamoxifen-infused rats to ER antagonism by 4-hydroxytamoxifen.

In addition, increased ER expression and decreased arginase activity were observed in both tamoxifen-infused and ovariectomized rats in this study. Increased ER levels may reflect a compensatory up-regulation under conditions of low estrogen or ER antagonism [19]. Arginase has been shown to modulate vascular responses in female genital tissue by decreasing intracellular arginine pools and inhibiting nitric oxide synthesis through substrate competition [34]. The decrease in arginase activity in tamoxifen-infused rats may be reflective of a compensatory down-regulation in the local vasculature to maintain vaginal perfusion. Alternatively, since arginase has been shown in other tissues to be critical for regulating cell growth via the polyamine synthesis pathway [35,36], the lower arginase activity may be an early physiological change that eventually results in vaginal atrophy with prolonged tamoxifen treatment. Future studies examining the time course of changes with tamoxifen treatment would be helpful in this regard. Longer periods (>2 weeks) of tamoxifen treatment may also result in structural changes in the lamina propria and muscularis layers that were not evident in this study. Nevertheless, the parallel changes observed in tamoxifen-infused and ovariectomized rats suggest that tamoxifen generally exerts an anti-estrogenic effect in the vagina.

Tamoxifen also exerted a pro-estrogenic effect in the vaginal epithelium. The thickened epithelium and the dramatic increase in PAS-positive secretory vesicles within the apical cells suggested a highly mucified epithelium in tamoxifen-infused animals. Since PAS stains all 1,2-glycol carbohydrates (glycogen, mucins and glycolipids) the specific identity of the secretory product(s) remained uncharacterized. It is also possible that tamoxifen may interfere with vaginal epithelial differentiation in the rat. While glycogen and mucin production are part of this process, post-translational modifications of keratins in the apical layer of the vaginal epithelium have also been shown to be regulated by estradiol [37,38]. At least one of these processes (transamidation) is inhibited by tamoxifen [37]. These post-translational modifications are critical for keratin filament formation and aggregation. Also, since ER regulates synthesis of the progesterone receptor (PR), it is possible that antagonism of ER by tamoxifen results in PR downregulation and that PR may be involved in exocytosis of glycogen from the vaginal epithelium. Thus, tamoxifen may inhibit the terminal differentiation of vaginal epithelial cells without interfering with intermediate differentiation processes.

It is interesting to note that the estrogenic effects of tamoxifen on the vaginal epithelium have been observed by other investigators using animal models [39-41]. Clinical studies have also consistently verified the estrogenic effects of tamoxifen on the vaginal epithelium [42-47]. However, in breast cancer patients treated with tamoxifen, although cytological examination of vaginal epithelial cells indicated a high maturation index, this estrogenic effect was unrelated to sexual function, duration of tamoxifen treatment or type of breast cancer surgery [7]. Thus, the relevance of tamoxifen's effect on the vaginal epithelium to sexual function remains unclear.

Some of the cell and tissue-specific effects of tamoxifen and its metabolites may be attributed to their multiple modes of action. In addition to being an ER antagonist, tamoxifen (and/or its metabolites) has been shown to be an inhibitor of protein kinase C and a calmodulin antagonist [48,49]. In general, these events lead to inhibition of cell growth and are presumably responsible for tamoxifen's ability to inhibit tumorigenesis. More importantly, the interaction of the tamoxifen-bound ER with various co-activators and co-repressors may be critical in determining the pro- or anti-estrogenic effects of tamoxifen [50-52]. Since different cell types may have different levels of protein expression for each co-regulator, the overall balance of co-regulatory proteins with activating and repressing activity may determine whether tamoxifen has an estrogenic or anti-estrogenic effect. In addition, a membrane protein with characteristics identical to ERα may stimulate the phosphoinositide pathway in rat vaginal epithelial cells, [53,54]. However, whether the epithelium is unique in this regard, relative to other cell types in the vagina, remains to be established.

A limitation of our study is that we did not investigate the effects of tamoxifen on testosterone metabolism or androgen receptors. Given that plasma testosterone and estradiol both decrease in ovariectomized animals, analogous changes between tamoxifen-treated and ovariectomized rats do not necessarily indicate that these changes are due entirely to estrogen-dependent mechanisms. It is possible that the effects of tamoxifen are partially dependent upon androgens. For example, in male rats, tamoxifen has been shown to decrease circulating levels of luteinizing hormone and testosterone and to increase liver enzymes that metabolize androgens [55,56]. In postmenopausal women with breast cancer, tamoxifen treatment resulted in increased sex hormone binding globulin and decreased levels of circulating androgens [57]. Further, some studies suggest that tamoxifen may directly bind the androgen receptor and can inhibit androgen receptor activity [58,59]. Thus, the effects of tamoxifen are complex and may be manifested through multiple and inter-related receptor systems.

It should be further emphasized that tamoxifen may exert effects that are species specific. Different responses may occur due to unique expression profiles of co-activator and co-repressor proteins. In addition, rats have been shown to metabolize tamoxifen to hydroxytamoxifen much more efficiently than humans [60]. Secondary metabolites produced by the action of sulfotransferases can also have mutagenic activity through the formation of DNA adducts. Rat hydroxysteroid sulfotransferase has been shown to have at least 20-fold greater ability to produce mutagenic metabolites when compared to the comparable human enzyme [61]. Thus, rats may exhibit a greater sensitivity to both the anti-estrogenic and mutagenic actions of tamoxifen than humans.

## Conclusion

Tamoxifen administration in the female rat causes functional changes in vaginal tissue that parallel the estrogen-deprived state. Such alterations may eventually lead to vaginal atrophy, dryness and pain, compromising sexual function. The apparent pro-estrogenic effect of tamoxifen on vaginal epithelium and its relevance to sexual function remains unclear, but is consistent with previous laboratory and clinical observations. Vaginal epithelial cells may manifest a unique expression profile of ER co-regulatory proteins, as well as membrane-associated and soluble ER species that enable tamoxifen to mediate an estrogenic effect on the epithelium while acting as an anti-estrogen on other cell types in the vagina. The molecular alterations responsible for the observed functional changes should be the subject of future studies examining the mixed effects of tamoxifen (and potentially other SERMs) on the vagina. Given the current widespread use of tamoxifen in the management of post-menopausal breast cancer patients, the prospect of increased use of tamoxifen or other SERMs in younger, premenopausal women at risk for breast cancer, and the importance of sexual health to the patient's quality of life, additional research on the effects of tamoxifen on genital organ function are warranted.

## Competing interests

The author(s) declare that they have no competing interests.

## Authors' contributions

NNK participated in the design of the study, assisted with the animal treatment protocols and data collection, analysed the data, prepared the figures, and maintained a lead role in writing and revising the manuscript. MS and AA carried out the animal treatment protocols, performed the *in vivo *blood flow studies, assisted in tissue collection and preliminary data analysis, and participated in revising the manuscript. TTC assisted in tissue collection, carried out all histological processing and evaluations (including estrous cycle staging), and participated in revising the manuscript. IG participated in conceiving the study and assisted with drafting and revising the manuscript. AMT assisted in conceiving and designing the study and participated in drafting and revising the manuscript. All authors have read and approved the final manuscript.

## Pre-publication history

The pre-publication history for this paper can be accessed here:


